# Activation of the UPR sensor ATF6α is regulated by its redox-dependent dimerization and ER retention by ERp18

**DOI:** 10.1073/pnas.2122657119

**Published:** 2022-03-14

**Authors:** Ojore Benedict Valentine Oka, Arvin Shedrach Pierre, Marie Anne Pringle, Wanida Tungkum, Zhenbo Cao, Bethany Fleming, Neil John Bulleid

**Affiliations:** ^a^Institute of Molecular, Cell and Systems Biology, College of Medical Veterinary and Life Sciences, University of Glasgow, Glasgow G12 8QQ, United Kingdom

**Keywords:** ER stress, unfolded protein response, ATF6, proteostasis

## Abstract

The unfolded protein response (UPR) maintains cellular proteostasis during stress by activating sensors located to the endoplasmic reticulum (ER) membrane. A major sensor for this response, ATF6α, is activated by release from ER retention and trafficking to the Golgi, where it is cleaved to generate a bZIP transactivator to initiate a transcriptional response. The reduction of a disulfide in monomeric ATF6α is thought to be necessary for release from retention, trafficking, and proteolysis. Here we show that, following ER stress, ATF6α undergoes a redox switch to form a disulfide bonded dimer, which traffics to the Golgi for cleavage by the S1P protease. Additionally, we find that overexpression of ERp18 attenuates dimer formation thereby limiting Golgi trafficking. Our results provide mechanistic insight into activation of the ATF6α pathway, revealing an unexpected role for redox-dependent oligomerization prior to Golgi trafficking.

## Significance

Membrane and secretory proteins are synthesized in the endoplasmic reticulum (ER). Perturbations to ER function disrupts protein folding, causing misfolded proteins to accumulate, a condition known as ER stress. Cells adapt to stress by activating the unfolded protein response (UPR), which ultimately restores proteostasis. A key player in the UPR response is ATF6α, which requires release from ER retention and modulation of its redox status during activation. Here, we report that ER stress promotes formation of a specific ATF6α dimer, which is preferentially trafficked to the Golgi for processing. We show that ERp18 regulates ATF6α by mitigating its dimerization and trafficking to the Golgi and identify redox-dependent oligomerization of ATF6α as a key mechanism regulating its function during the UPR.

The lumen of the endoplasmic reticulum (ER) contains numerous proteins and enzymes that ensure timely and efficient folding of proteins entering the secretory pathway (1). Cellular insults and disruptions to ER function often result in misfolding and inefficient trafficking of proteins, leading to ER stress and an unfolded protein response (UPR) (2). The UPR is accomplished by activation of three transmembrane transducers localized to the ER: namely, IRE1α, PERK, and ATF6α (3). Activation of the UPR triggers the up-regulation of genes encoding for folding, degradation, and quality control factors, as well as the selective reduction of protein translation (3, 4), which ultimately restores proteostasis and normal ER function.

IRE1 is the most conserved of the UPR pathways. IRE1 activation promotes its cytosolic kinase and ribonuclease activities (5, 6), which ultimately results in splicing of XBP1 mRNA to produce an active transcription factor (7). XBP1 up-regulates the expression of ER folding and quality-control proteins. The nuclease activity of IRE1 also mediates regulated IRE1-dependent mRNA decay that promotes rapid turnover of mRNAs encoding membrane and secreted proteins (8). PERK activation leads to phosphorylation of eIF2α, which promotes attenuation of global protein translation (9), while selectively promoting translation of ATF4 transcription factor. ATF4 regulates numerous genes important in cellular adaptation to stress (10, 11). However, under conditions of prolonged and sustained ER stress, ATF4 activates the CHOP pathway to initiate apoptosis, thereby regulating both prosurvival and procell death processes (3).

The mechanism of ATF6α activation is less well understood. During ER stress, ATF6α traffics to the Golgi apparatus, where it is sequentially cleaved by site-1 protease (S1P) and site-2 protease (S2P) to release a soluble bZIP transcription factor, ATF6-N (12). ATF6-N translocates to the nucleus and transactivates UPR genes encoding for ER folding and ERAD factors (13, 14). Regulation of ATF6α is afforded by ER retention, by BiP and ERp18 (15, 16), trafficking to the Golgi via COPII-coated vesicles (17, 18) and processing by the Golgi-localized proteolytic enzymes S1P and S2P (12). One of the well-characterized ER retention factors, BiP, contains multiple binding sites on ATF6α, including two potential Golgi localization sequences (15, 19). Early events during ER stress cause BiP to be released from ATF6, allowing it to package into COPII vesicles. What promotes BiP release from ATF6α remains unclear; however, it has been proposed that competition for binding to misfolded proteins is involved (20).

In unstressed cells, ATF6α exists as three redox forms, a monomer and two interchain disulfide-stabilized dimers. These redox forms are maintained by the presence of two highly conserved cysteines in the luminal domain, C467 and C618 (21). These cysteines engage in either an intramolecular disulfide or form interchain disulfides between C467-C467 and C618-C618 to form two distinct ATF6α dimers (21). The redox and oligomeric status of ATF6α has been reported to regulate its trafficking and proteolysis in the Golgi (21, 22). It was reported that during ER stress ATF6α disulfides are modified to form reduced monomeric ATF6α that specifically traffics to the Golgi and is the preferred substrate for S1P cleavage (21). Hence, the reduction of disulfides in ATF6α is thought to occur prior to Golgi trafficking. Here we investigated changes to the redox status and dimerization of ATF6α during ER stress induced by both chemotoxic and proteotoxic stress. We report that the ATF6α C467 dimer and not the monomer specifically accumulates during the early stages of the UPR and that the C467 dimer is preferentially transported to the Golgi and processed by S1P and S2P to produce soluble ATF6-N. ERp18 acts to retain ATF6α in the ER, thereby regulating packaging into transport vesicles. Our results highlight a previously unexpected role for interchain disulfide formation in the activation of ATF6α and establishes dimerization as a prerequisite to Golgi trafficking.

## Results

### ER Stress Promotes Redox-Dependent Formation of a Hyperglycosylated ATF6α C467 Dimer.

**Fig. 1.**
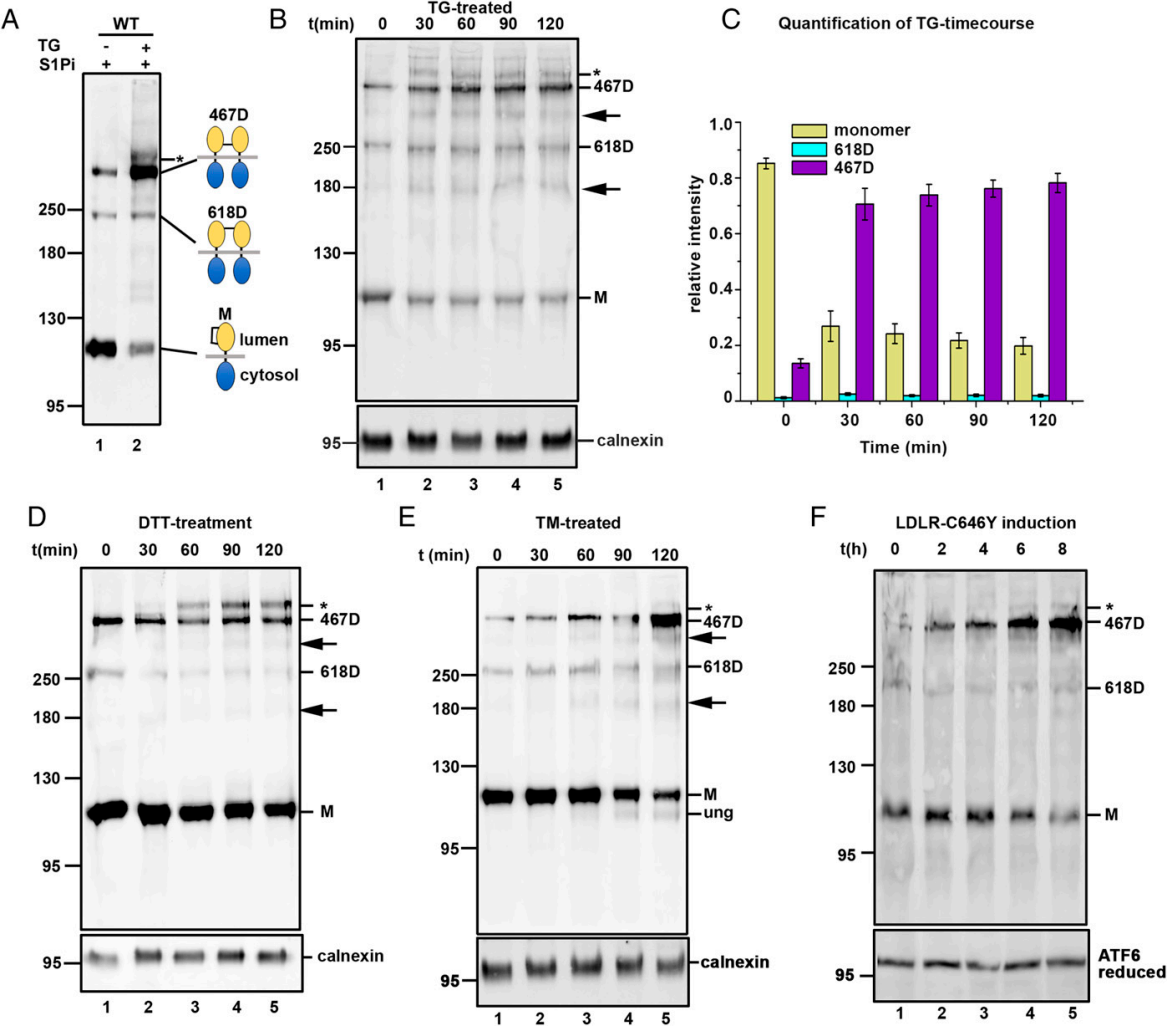
ER stress promotes accumulation of ATF6α C467 dimer. (*A*) Wild-type ATF6α overexpressing HEK293T cells (HEK/ATF6), were pretreated with 30 µM S1Pi for 60 min, and then either untreated (−) or treated (+) with 5 µM TG for 90 min. Cell lysates were separated under nonreducing SDS/PAGE conditions followed by rabbit anti-HA Western blotting to detect ATF6α. The schematic shows the C467 dimer (467D), C618 dimer (618D) and monomer ATF6α (M) including their inter- or intramolecular disulfide. Yellow and blue spheres represent ATF6α luminal domain and cytosolic region, respectively. Asterisks indicates hyperglycosylated 467D. (*B*) HEK/ATF6 cells were pretreated with 30 µM S1Pi for 60 min, then with 5 µM TG for the indicated times. Cell lysates were separated under nonreducing SDS/PAGE conditions followed by mouse anti-V5 and rabbit anticalnexin Western blots. Arrows indicate uncharacterized V5-reactive bands. (*C*) The percentage intensities of the various ATF6α forms from *B*, were quantified, error bars represent ± SD for three independent experiments. (*D* and *E*) HEK/ATF6 cells were pretreated with 30 µM S1Pi and with 1 mM DTT or 5 µg tunicamycin (TM), respectively, for the indicated times. Cell lysates were separated under nonreducing SDS/PAGE conditions and ATF6α detected by mouse anti-V5 and rabbit anticalnexin Western blots. Underglycosylated ATF6α in TM-treated cells indicated as “ung.” Arrows indicate uncharacterized V5- reactive bands. (*F*) Flp-In T-Rex doxycycline-inducible HeLa cells expressing HA-tagged mutant lipoprotein receptor C646Y (HA-LDLR C646Y) were pretreated with the S1Pi, then treated with 2 μg/mL doxycycline for indicated times. Cell lysates were separated under reducing and nonreducing SDS/PAGE conditions and ATF6 detected with mouse anti-ATF6α.

The ATF6α luminal domain contains two cysteine residues (C467 and C618) that either form an intrachain disulfide (C467-618) in the monomer or interchain disulfide bonded dimers (C467-467 or C618-618) (16) (Fig. 1*A*). To investigate any changes to the redox status of ATF6α during ER stress, we used a previously characterized HEK293T cell line stably expressing epitope tagged ATF6α containing a HA-tag in the cytosolic and a V5-tag in the luminal domain (16). To prevent ATF6α cleavage by S1P during ER stress, cells were treated with an S1P inhibitor (S1Pi) (23) before treatment with various stressors. We have shown previously that S1P inhibition prior to induction of ER stress prevents ATF6α cleavage allowing it to accumulate in the Golgi and become hyperglycosylated (16). Initially, we analyzed the redox status of ATF6α after chemotoxic stress induced by thapsigargin (TG). When cell lysates were separated under nonreducing SDS/PAGE conditions and immunoblotted with anti-HA, we detected three bands: ATF6α monomer (designated M) and two dimers, designated 618D and 467D (Fig. 1*A*), as reported previously (16, 21). In unstressed cells, ATF6α consisted mainly of the monomer (Fig. 1*A*, lane 1). However, following ER stress, the intensity of the ATF6α monomer becomes significantly reduced, while the intensity of the ATF6α 467D increased to become the predominant form (Fig. 1*A*, lane 2). The ATF6α 618D remained mostly unchanged. Furthermore, in the TG-treated cells, we detected a slower-migrating band above the ATF6α 467D, (indicated with an asterisk in Fig. 1*A*), which was absent in unstressed cells. This band indicates further glycosylation modification of ATF6α 467D, consistent with Golgi-localization. We found the same redox change and hyperglycosylation with endogenous ATF6α visualized with an ATF6α-specific antibody (*SI Appendix*, Fig. S1) even at 10-fold lower concentrations of added TG, indicating the effects were not specific to ectopically expressed protein or to the TG concentration used to induce the UPR.

Previous reports have shown that hyperglycosylation of ATF6α occurs during ER stress, which results in reduced SDS/PAGE mobility (15, 16). We carried out a time course of TG treatment to follow the kinetics of accumulation of ATF6α 467D and its subsequent hyperglycosylation (Fig. 1*B*). After 30 min, we observed a reduction in the intensity of the ATF6α monomer and an increase of the ATF6α 467D, including the appearance of the hyperglycosylated ATF6α 467D (Fig. 1*B*, lane 2). Additional faint bands were detected between the 467D, 618D, and monomer with increased intensity after TG treatment (depicted here and in subsequent experiments where apparent with arrowheads). These varied in intensity between experiments, were particularly apparent when the V5-antibody was used, and were seen in the presence of S1Pi, suggesting they are not generated due to S1P cleavage. They may represent alternate redox forms of ATF6α and require further investigation for their identification. Over 70% of the ATF6α detected after 30 min of TG treatment was ATF6α 467D (Fig. 1*C*), and this level remained unchanged for the duration of the time course (Fig. 1*B*, lanes 2 to 5, and Fig. 1*C*). The intensities of the ATF6α 618D remained mostly unaffected (Fig. 1*B*, lanes 1 to 5, and Fig. 1*C*). These results demonstrate that the redox status of ATF6α changes following chemotoxic stress, consistent with a reduction of the C467-C618 disulfide in the monomer and formation of a C467-467 disulfide bonded dimer.

To examine the redox changes to ATF6α 467D following ER stress induced by different reagents, we carried out time courses in the presence of dithiothreitol (DTT), which disrupts protein disulfide formation, or tunicamycin, a glycosylation inhibitor. After 30 min of DTT treatment, we did not detect any significant changes in the levels of ATF6α monomer and 467D; however, we detected the appearance of the hyperglycosylated form of 467D (Fig. 1*D*, lane 2). The hyperglycosylated ATF6α 467D reached its peak after 90 min and was still detectable after 120 min (Fig. 1*D*, lanes 4 and 5). The apparent lack of an increase in the levels of ATF6α 467D after 30 min of DTT treatment might be due to reduction of the interchain disulfide by DTT itself or the rapid conversion of ATF6α 467D to the hyperglycosylated form. At higher concentrations, DTT can reduce the ATF6α intra- and intermolecular disulfides (21) (*SI Appendix*, Fig. S2). Here we used a relatively low concentration of DTT to minimize ATF6α disulfide reduction but still allow a robust UPR. Even in the presence of the reducing agent, the main species that becomes hyperglycosylated is 467D.

In tunicamycin-treated cells, we also detected a decrease in the ATF6α monomer with a concomitant increase in the 467D, although the accumulation of 467D in these cells exhibited a slower appearance compared to the TG-treated cells (compare Fig. 1 *B* and *E*). This is consistent with tunicamycin displaying a slower kinetics of activation of ATF6α compared with TG (21). We also detected unglycosylated ATF6α (ung) after 90 and 120 min (Fig. 1*E*, lanes 4 and 5), consistent with tunicamycin inhibition (21, 24). At the later time point, some hyperglycosylated 467D is apparent (Fig. 1*E*, lane 5). Hence, for the chemotoxic agents studied that induce ER stress there is a specific hyperglycosylation of the 467D, suggesting that this form is selectively transported to the Golgi.

The commonly used UPR inducers, DTT, TG, and tunicamycin exert multiple effects on cellular homeostasis, in addition to activating the UPR pathways. It is apparent that chemical induction of the UPR confers different genetic and protein expression signatures in comparison to the UPR induced by expression of misfolded proteins in the ER (25). To investigate changes to the redox status of ATF6α under proteotoxic induction of the UPR, we generated a Flp-In T-Rex doxycycline-inducible HeLa cell line for expression of HA-tagged mutant lipoprotein receptor (LDLR), HA-LDLR C646Y. This mutation causes a defect in LDLR folding and trafficking and consequently the misfolded protein accumulates in the ER (26, 27). To investigate if expression of misfolded HA-LDLR C646Y would elicit a UPR response, cells were treated with doxycycline for various times and HA-tagged LDLR was detected by immunoblot using anti-HA. Expression of HA-LDLR was seen after 2 h, with a steady increase over the time course (*SI Appendix*, Fig. S3). Immunoblot analyses of endogenous ATF6α in these cells revealed the presence of two forms, monomer and 467D, with the 618D, barely detectable, mirroring that seen with exogenously expressed ATF6α (Fig. 1 *A* and *F*). Interestingly, there was a shift in the redox status of ATF6α from predominantly the monomer to the 467 dimer, with maximal levels of the dimer seen at 8 h (Fig. 1*F*, lane 5). We also detected some hyperglycosylation of the 467D at later time points (Fig. 1*F*, lanes 4 and 5). Note the time course of induction was carried out in the presence of S1Pi to prevent ATF6α processing. The change from the monomer to the 467D coincides with the increase in expression of the misfolded HA-LDLR (*SI Appendix*, Fig. S3), suggesting that accumulation of this protein in the ER is sufficient to induce a change in the redox status of ATF6α. The expression of misfolded LDLR activated the IRE1 pathway, as indicated by the splicing of XBP1-u to produce XBP1-s, thereby confirming a UPR response (*SI Appendix*, Fig. S4). In addition, levels of BiP increased after induction of misfolded LDLR in the absence of S1Pi (*SI Appendix*, Fig. S3*A*). This increase was attenuated in the presence of S1Pi (*SI Appendix*, Fig. S3*B*). Importantly, the predominant form of ATF6α during ER stress produced by proteotoxic stress is the 467D, which becomes hyperglycosylated indicating selective transport to the Golgi. These results are consistent with our finding with chemotoxic stressors and confirm that the redox changes seen with exogenously expressed ATF6α also occur with endogenous ATF6α.

### ATF6α 467D Is Preferentially Trafficked to the Golgi.

**Fig. 2.**
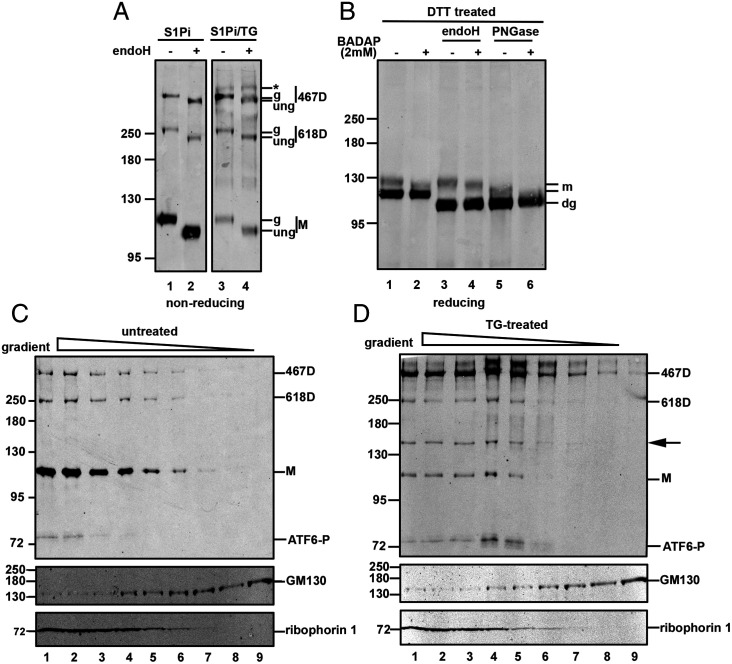
ATF6α 467D is preferentially transported to the Golgi during ER stress. (*A*) Wild-type HEK/ATF6 cells were pretreated with 30 µM S1Pi for 60 min, then either untreated (lanes 1 and 2) or treated with 5 µM TG for 90 min (lanes 3 and 4). Cell lysates were either left untreated or incubated with endoglycosidase H (endoH) as indicated, separated under nonreducing SDS/PAGE followed by rabbit anti-HA Western blot to detect ATF6α. Asterisk, g, and ung indicate the respective hyperglycosylated, glycosylated, and deglycosylated forms of ATF6α. (*B*) HEK/ATF6 cells, were untreated (−) or treated (+) with 2 mM BADAP and 30 µM S1Pi for 60 min. The cells were then treated with 10 mM DTT. Cell lysates were left undigested (lanes 1 and 2) or digested with endoH (lanes 3 and 4) or Peptide: *N*-glycosidase F (PNGase) (lanes 5 and 6). Samples were separated under reducing SDS/PAGE conditions and ATF6α detected using rabbit anti-HA. The reduced monomer and its hyperglycosylated forms are indicated (m), and “dg” represents deglycosylated form of ATF6α. (*C*) HEK/ATF6 cells were subject to sucrose gradient fractionation and samples separated under nonreducing SDS/PAGE condition. ATF6α was detected by rabbit anti-HA Western blot. Ribophorin I and GM130 were detected with rabbit anti-ribophorin I and rabbit anti-GM130, respectively. The appearance of the ATF6 cleavage product is indicated (ATP6-P). (*D*). As described in *C*, however, cells were pretreated with 30 µM S1Pi for 60 min, and then with 5 µM TG for 90 min before fractionation.

The ER stress induced hyperglycosylation of ATF6α 467D would suggest that it is transported from the ER to the Golgi during the UPR. To further elucidate the location of ATF6α 467D hyperglycosylation, we analyzed the sensitivity of ATF6α monomer and dimers to endoglycosidase H (endo H) digestion. Cells pretreated with S1Pi were left untreated or treated with TG, to induce ER stress and cell lysates incubated with or without endo H. In unstressed cells, ATF6α monomer and dimers were sensitive to endo H digestion, as demonstrated by their increased SDS/PAGE mobilities (Fig. 2*A*, lane 2), indicating ER localization. Only the hyperglycosylated C467 dimeric form generated after ER stress was resistant to endo H digestion, consistent with trafficking to the Golgi (compare lanes 3 and 4, band with asterisk, in Fig. 2*A*) (15).

To determine if hyperglycosylated ATF6α 467D is O-linked glycosylated, we treated cells with the O-linked glycosylation inhibitor, benzyl-2-acetamido-2-deoxy-α-d-galactopyranoside (BADAP) (28, 29). Cells were pretreated with S1Pi, and then with 10 mM DTT for 30 min. We favored a high DTT concentration as the stressor for this experiment since it causes a robust activation of ATF6α and results in a maximal build-up of hyperglycosylated ATF6α (16) (*SI Appendix*, Fig. S2). Treatment with BADAP resulted in increased SDS/PAGE mobilities of the hyperglycosylated ATF6α before or after endo H treatment suggesting inhibition of O-linked glycosylation (Fig. 2*B*, lanes 1 to 4). Additionally, whereas hyperglycosylated ATF6α was resistant to endo H digestion (Fig. 2*B*, lanes 1 and 3), incubation with PNGase resulted in increased mobility due to removal of N-linked oligosaccharides (Fig. 2*B*, lanes 1 and 5). Interestingly, treatment with BADAP followed by PNGase caused a further increased mobility of hyperglycosylated ATF6α (Fig. 2*B*, lane 6). Together, these results demonstrate the reduced mobility form of ATF6α has modifications to the N-linked glycans conferring endo H resistance as well as O-linked glycosylation that can be inhibited by BADAP.

Previously, it was suggested that only monomeric ATF6α traffics to the Golgi based upon stress induction with DTT or tunicamycin, followed by fractionation of the ER and Golgi by discontinuous sucrose gradient. We also fractionated lysates from unstressed and stressed cells in a discontinuous sucrose density gradient, to partially separate ER and Golgi components. In unstressed cells, all forms of ATF6α were detected in the heavier fractions (Fig. 2*C*, lanes 1 to 5), consistent with its ER localization. Western blotting demonstrated the presence of the ER membrane protein, ribophorin, in fractions 1 to 5, confirming the presence of ER components in the heavier fractions. GM130, used as a Golgi marker, partially overlaps with ribophorin, but was detected primarily in the lighter fractions (Fig. 2*C*, lanes 4 to 9). In the TG-treated cells, we also detected ATF6α monomer and dimers in the heavier ER fractions (Fig. 2*D*, lanes 1 to 5). The hyperglycosylated 467D was predominantly found in lighter fractions (Fig. 2*D*, lanes 4 and 5). Note the presence of a cleaved from of ATF6α (ATF6-P) that migrates at about 75 kDa that we have previously reported and is often seen after treatment with the S1Pi (16). Sucrose gradient fractionation of cells treated with tunicamycin also showed that hyperglycosylated ATF6α 467D transitioned to lighter fractions (*SI Appendix*, Fig. S5, lanes 4 and 5). These results indicate stress-induced trafficking of ATF6α 467D from ER fractions to lighter fractions consistent with the early Golgi compartment.

**Fig. 3.**
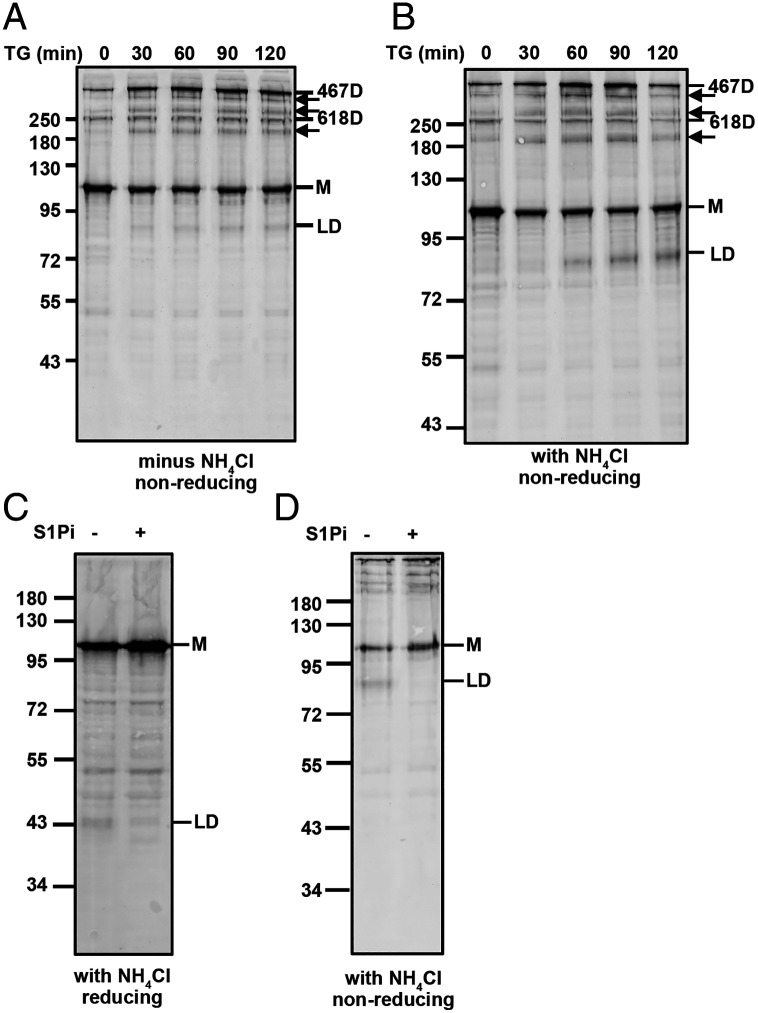
ER stress induced cleavage of ATF6α produces a luminal domain (LD) dimer. (*A* and *B*) HEK/ATF6 cells, were untreated *(A*) or treated (*B*) with 20 mM ammonium chloride (NH_4_Cl) for 45 min, and then with 5 µM TG, for the indicated times. Cell lysates were separated under nonreducing SDS/PAGE conditions and ATF6α detected with mouse anti-V5. The luminal domain dimer is indicated (LD). Arrows indicate uncharacterized V5-reactive bands. (*C* and *D*) HEK/ATF6 cells were treated with 20 mM NH_4_Cl, in the absence (−) or presence (+) of 30 µM S1Pi for 60 min. The 5 µM TG treatment was carried out for 60 min. Whole-cell lysates were separated under reducing (*C*) or nonreducing (*D*) SDS/PAGE conditions, followed by mouse anti-V5 blots.

The trafficking of ATF6α 467D from the ER to Golgi should result in the formation of a disulfide bonded luminal domain dimer following cleavage by S1P. To determine if this was the case, we immunoblotted with anti-V5, cell lysates from cells treated with TG in the absence of the S1Pi (Fig. 3). The ATF6α construct contains a V5-epitope at the C terminus allowing detection of any cleaved luminal domain. After 30 min of TG treatment, a V5 reactive band was detected migrating at about 90 kDa (Fig. 3*A*). When cells were pretreated with ammonium chloride, before treatment with TG, the intensity of the ATF6α luminal domain dimer was increased (Fig. 3*B* and *SI Appendix*, Fig. S6*A*), suggesting that inhibition of lysosomal degradation (30, 31) stabilizes the cleaved ATF6α luminal domain. Similar stabilization of the luminal domain was seen in the presence of another lysosomal degradation inhibitor, Bafilomycin A1 (*SI Appendix*, Fig. S6*A*). This band was absent when the samples were immunoblotted with an anti-HA antibody (*SI Appendix*, Fig. S6*B*) whose epitope is located at the N-terminal cytosolic region of ATF6α. We confirmed this band to be a result of cleavage by S1P, as it was undetected in the presence of S1Pi (Fig. 3 *C* and *D* and *SI Appendix*, Fig. S6*A*) and migrated at about 45 kDa when samples were analyzed under reducing SDS/PAGE (Fig. 3*C*). No monomeric luminal domain was detected during the time course of TG treatment (Fig. 3 *A* and *B*), suggesting that dimerization occurs prior to Golgi trafficking and cleavage. These results demonstrate that ATF6α 467D is preferentially trafficked to the Golgi during stress and suggest that the released ATF6α luminal domain localizes to the lysosome for degradation.

### Role of Interchain Disulfide Formation in ATF6 Trafficking.

**Fig. 4.**
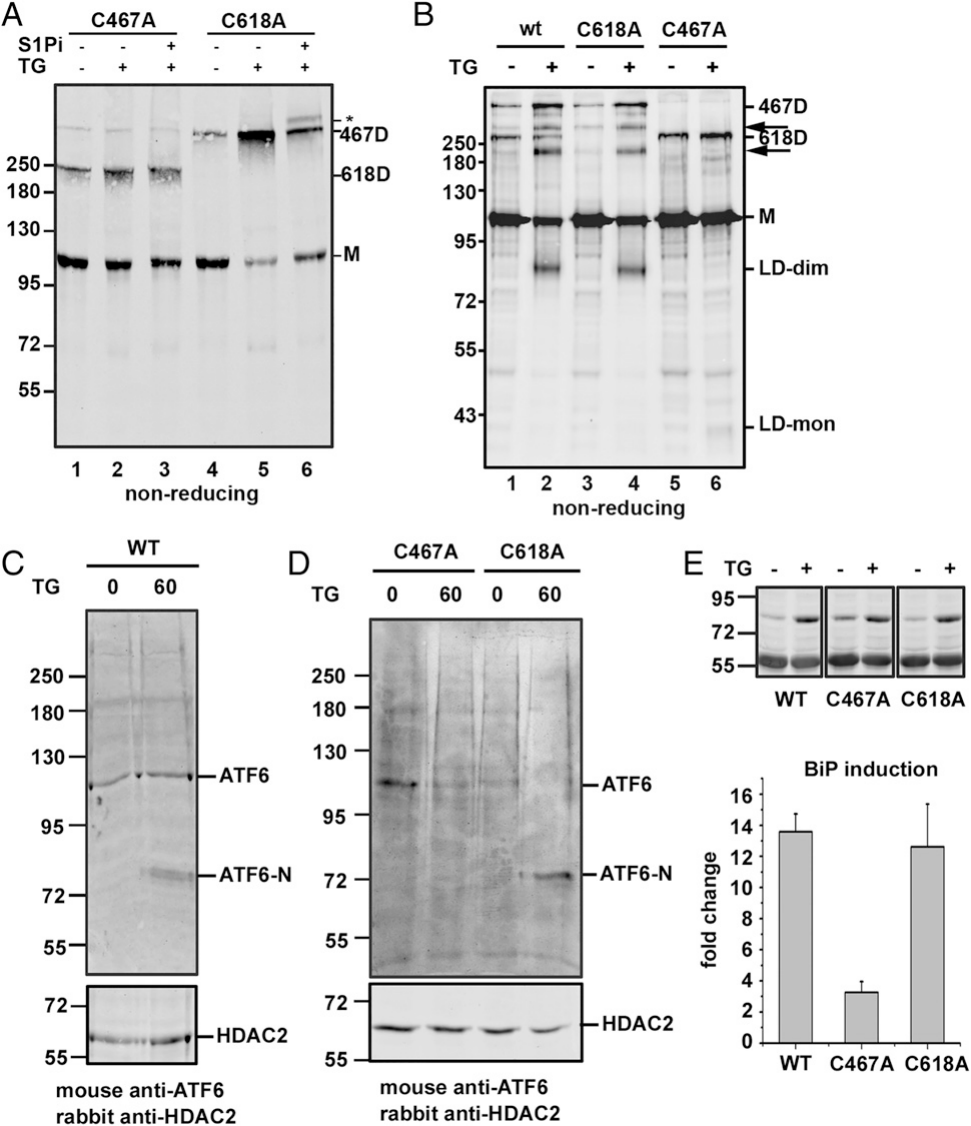
Analyses of ATF6 luminal domain cysteine mutants. (*A*) ATF6α-deficient HT1080 cells (HT1080/ATF6α KO) stably overexpressing ATF6α C467A and C618A were either untreated (−) or treated (+) with the S1Pi and TG as indicated. Whole-cell lysates were separated under nonreducing SDS/PAGE conditions and ATF6α detected using mouse anti-ATF6. (*B*) HT1080/ATF6 KO cells were transiently transfected with the wild-type, C618A, and C467A mutants of ATF6α. Cells were treated with ammonium chloride and TG as indicated. Samples were separated under nonreducing SDS/PAGE, followed by anti-V5 Western blot. LD-dim and LD-mon represent the ATF6α luminal domain dimer and monomer respectively. Arrows indicate uncharacterized V5-reactive bands. (*C* and *D*) HT1080/ATF6α KO cells stably overexpressing ATF6α wild-type (WT), C467A, and C618A mutants, were treated with TG as indicated. Cells were then subject to differential centrifugation to obtain the nuclear fractions, which were separated under reducing SDS/PAGE conditions and analyzed by mouse anti-ATF6α and rabbit anti-HDAC2 Western blots. (*E*) HT1080/ATF6α KO cells stably overexpressing ATF6α wild-type, C467A, and C618A mutants were left untreated or treated with 1 µM TG for 16 h. BiP fold-change were quantified from lysates separated under reducing SDS/PAGE and BiP levels detected by Western blotting. Error bars represent ± SD for three independent experiments.

To determine the potential role of the interchain disulfides within the ATF6α luminal domain in activation and trafficking, we generated stable cells individually expressing ATF6α C467A and C618A mutants in a HT1080 fibrosarcoma cell line where the ATF6α gene was deleted. We used cells lacking endogenous ATF6α for these experiments to exclude the possibility of dimerization between endogenous and exogenously expressed ATF6α, which could lead to artifactual results. We confirmed the knockout (KO) of endogenous ATF6α in these cells (*SI Appendix*, Fig. S7) by immunoblot analyses (*SI Appendix*, Fig. S7*A*) and by comparing the induction of BiP expression upon TG-induced ER stress with wild-type cells (*SI Appendix*, Fig. S7 *B* and *C*). Cell lysates from either the C467A or C618A cells were treated with or without TG and separated under nonreducing SDS/PAGE (Fig. 4*A*). Immunoblotting with anti-ATF6α revealed the presence of monomers and dimers in unstressed cells (Fig. 4*A*, lanes 1 and 4), with the expected mobilities of the dimeric forms as previously reported (16, 21). Treatment with TG led to hyperglycosylation of the 467D but not the 618D (Fig. 4*A*, lanes 3 and 6). No hyperglycosylation was seen after TG treatment in the absence of S1Pi (Fig. 4*A*, lanes 2 and 5), indicating that trafficked ATF6α was rapidly cleaved in the Golgi. These results suggest that for the wild-type ATF6α and C618A mutant, it is the 467D form that is preferentially transported to the Golgi during ER stress where it becomes hyperglycosylated.

To identify which dimeric form of ATF6α gives rise to the luminal domain dimer, we transiently transfected the ATF6α KO cell line with wild-type, the C618A or C467A mutants of ATF6α, and determined the presence of the luminal domain dimer after ER stress and stabilization with ammonium chloride (Fig. 4*B*). The luminal domain dimer was only observed with the wild-type and C618A mutant after ER stress (Fig. 4*B*, lanes 2 and 4), but not the C467A mutant, though a small amount of monomeric luminal domain was observed (Fig. 4*B*, lane 6). These experiments with the cysteine mutants of ATF6α, further substantiate that 467D predominates after ER stress and it is this form of ATF6 that is preferentially transported to the Golgi.

To determine the functional consequence of this preferential transport, we also determined whether preventing formation of 467D would affect ATF6α cleavage or transcriptional activation. The cleavage of wild-type and the cysteine mutants of ATF6 was determined by preparing a nuclear extract and evaluating the appearance of the cleaved nuclear form of ATF6 (ATF6-N) (Fig. 4 *C* and *D*). For these experiments we used stable cell lines expressing exogenous ATF6α in the ATF6-KO cell line. Upon TG activation, ATF6-N was formed after 60 min with both the wild-type (Fig. 4*C*) and the C618A mutant, but not the C467A mutant (Fig. 4*D*). In addition, there was an attenuation of induction of the ATF6α target BiP with the C467A mutant, but the induction of expression of BiP with the C618A mutant was equivalent to that seen with the wild-type after TG treatment (Fig. 4*E*). The presence of ATF6-N and induction of BiP verifies trafficking and cleavage of the 467D in the Golgi, confirming the results with hyperglycosylation and sucrose gradient fractionation.

### Specificity of S1P for the Redox Forms of ATF6.

**Fig. 5.**
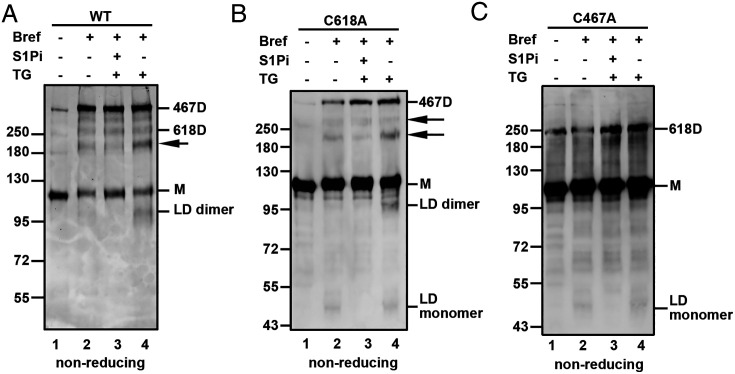
ATF6α redox status determines its S1P specificity. HT1080/ATF6 KO cells stably overexpressing wild-type ATF6α (*A*) and luminal domain cysteine mutants C618A (*B*) and C467A (*C*), were either untreated (−) or treated (+) with brefeldin A (Bref), S1Pi and TG as indicated. Cell lysates were separated under nonreducing SDS/PAGE conditions and ATF6α detected by mouse anti-V5 Western blots. Arrows indicate uncharacterized V5-reactive bands.

The presence of the S1P-generated luminal domain dimer with wild-type and C618A mutant, but not the C467A mutant of ATF6α, could be caused by the inability of S1P to cleave the 618D. To determine whether this was the case, we treated cells with brefeldin A and determined the appearance of the ATF6α luminal domain before or after ER stress (Fig. 5). Brefeldin A causes a merger of ER and Golgi components (32) and results in the cleavage of ATF6α by S1P and S2P in the absence of additional ER stress (16, 21). Lysates from brefeldin A-treated cells were separated under nonreducing SDS/PAGE followed by V5 immunoblot analysis. In wild-type ATF6α cells (Fig. 5*A*), treatment with brefeldin A alone did not result in any detectable cleavage product of ATF6α (Fig. 5*A*, lane 2). However, in the presence of TG and brefeldin A, we detected a dimeric ATF6α luminal domain (Fig. 5*A*, lane 4). The ATF6α luminal domain was absent in cells treated with S1Pi prior to brefeldin A and TG treatments (Fig. 5*A*, lane 3), confirming that its appearance was dependent upon S1P activity. The fact that brefeldin A treatment alone did not yield any detectable monomeric ATF6α luminal domain would suggest that the monomer present in these conditions is resistant to cleavage. TG treatment led to a switch from an intra- to an interchain disulfide resulting in enhanced S1P cleavage of ATF6α. In contrast, in the ATF6α C618A cells, treatment with brefeldin A alone resulted in a cleavage product of about 45 kDa, consistent with the ATF6α luminal domain monomer (Fig. 5*B*, lane 2). No intrachain disulfide can form with this mutant indicating the ability of S1P to cleave the reduced monomer. Interestingly, treatment of these cells with TG and brefeldin A led to the production of both monomer and dimeric forms of the ATF6α luminal domain (Fig. 5*B*, lane 4); these cleavage products were undetected in the presence of the S1Pi (Fig. 5*B*, lane 3). This result confirms that the 467D can be cleaved by S1P. In the ATF6α C467A cells, treatment with either brefeldin alone or in combination with TG resulted in the production of a monomeric ATF6α luminal domain (Fig. 5*C*, lanes 2 and 4), suggesting that the 618D is not a substrate for S1P. These results indicate that the redox status of ATF6α regulates the specificity of S1P, which preferentially cleaves the 467D or reduced monomer.

### ERp18 but Not PDIR Reduces the C467 Dimer and Promotes Retention of ATF6α in the ER.

**Fig. 6.**
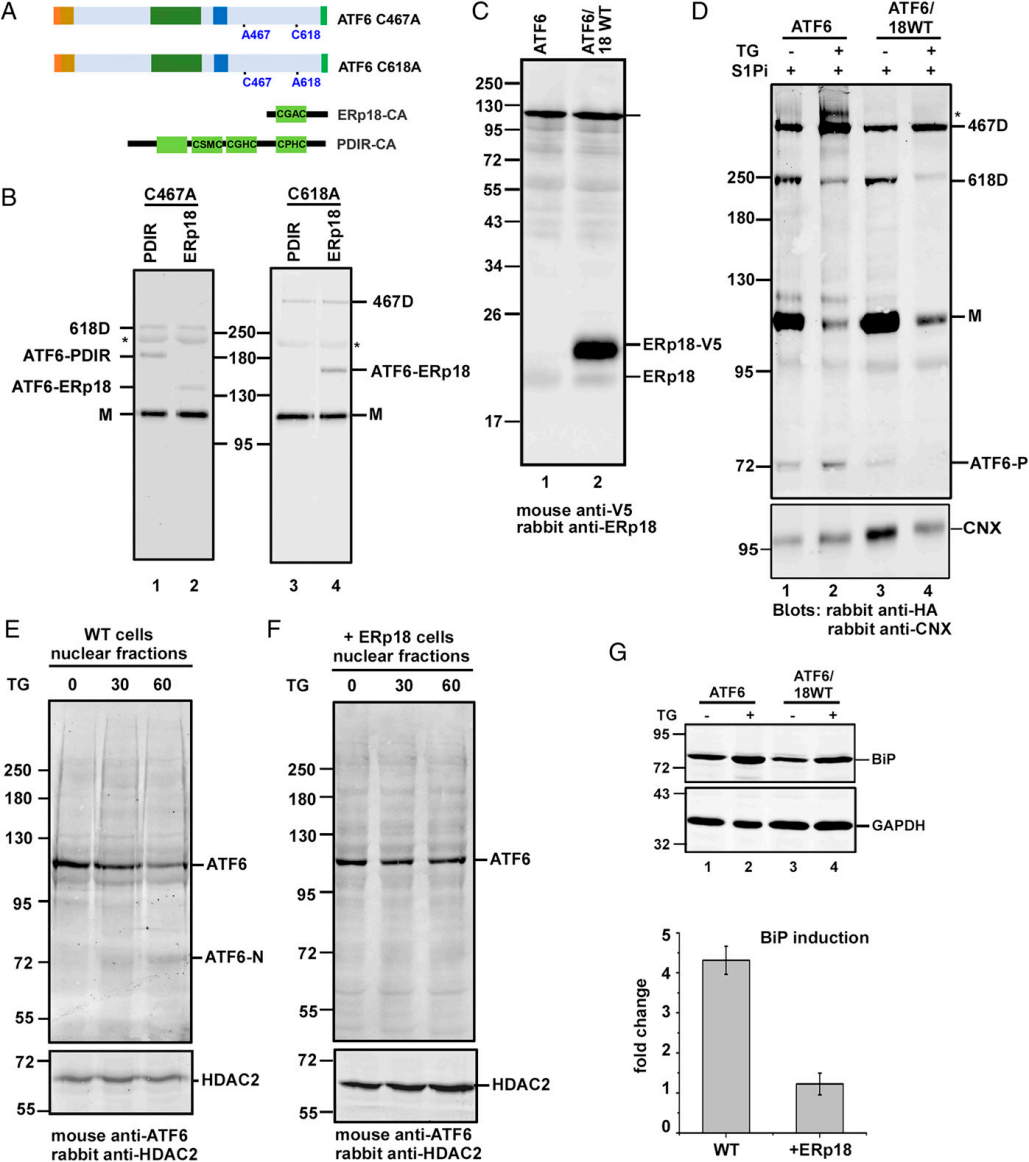
ERp18 promotes ATF6α retention in the ER. (*A*) Schematics of the ATF6α luminal domain cysteine mutants and the substrate-trapping mutants of the PDI oxidoreductases, ERp18 and PDIR, used in *B*. (*B*) HEK/ATF6 cells stably expressing the C467A and C618A mutants were transiently transfected with the substrate-trapping mutants of PDIR and ERp18. Twenty-four hours posttransfection, whole-cell lysates were immunoprecipitated with mouse anti-ATF6α, separated under nonreducing SDS/PAGE conditions, followed by Western blotting using rabbit anti-HA. Asterisks indicate background antibody band. (*C*) HEK/ATF6 cells and HEK/ATF6 cells stably expressing V5-tagged ERp18 (ERp18-V5) were analyzed by reducing SDS/PAGE, followed by Western blotting with mouse anti-V5 and rabbit anti-ERp18. (*D*) Cells from *C* were pretreated with the 30 µM S1Pi for 60 min, then with or without 5 µM TG as indicated for 90 min. Samples were separated under nonreducing SDS/PAGE, followed by rabbit anti-HA and rabbit anticalnexin Western blots. (*E* and *F*) Cells were treated with TG as indicated, then subjected to differential centrifugation to obtain the nuclear fractions. Samples were separated under reducing SDS/PAGE conditions and analyzed by mouse anti-ATF6α and rabbit anti-HDAC2 Western blots. The position of the ATF6 nuclear form (ATF6-N) is as indicated. (*G*) Cells were left untreated or treated with 1 µM TG for 16 h and the BiP fold-change was quantified from Western blots. Error bars represent ± SD for three independent experiments.

We have previously shown that ERp18 and protein disulfide isomerase-related (PDIR) are catalytically active toward ATF6α (16). We demonstrated that mutants of ERp18 or PDIR lacking the second cysteine in the CXXC active site can form mixed disulfides with ATF6α, demonstrating their ability to catalyze disulfide reduction (33). PDIR also has been implicated in the process of ATF6α activation (18), and is known to catalyze reduction of ATF6 disulfides in the absence of ER stress (16). As ATF6α switched its redox status following stress from the monomer to the 418D, we assessed here the specificity of these two enzymes toward either the 467-467 or 618-618 disulfide. When the substrate-trapping mutant of PDIR (Fig. 6*A*) was expressed in the presence of the C467A or C618A ATF6α mutants, mixed disulfides were seen with the C467A but not the C618A mutant (Fig. 6*B*, lanes 1 and 3). In contrast, the ERp18 substrate-trapping mutant formed mixed disulfides with both ATF6α mutants (Fig. 6*B*, lanes 2 and 4). These results indicate that ERp18 can reduce both disulfides, whereas PDIR specifically reduces the 618-618 disulfide. Indeed, we have previously shown that during ER stress, ERp18 can catalyze the reduction of the 467-467 disulfide indicating this enzyme antagonizes 467D formation.

Given the ability of ERp18 to reduce the 467-467 disulfide, we questioned whether overexpression of this oxidoreductase would lead to an attenuation of ATF6α activation. Our previous work had highlighted the role of ERp18 in regulating trafficking of ATF6α (16). Deletion of ERp18 resulted in faster trafficking of ATF6α due to a physical interaction between these two proteins following ER stress, indicating a role for ERp18 in retention. Therefore, we investigated the consequence of overexpression of ERp18 on both dimer formation, Golgi trafficking, S1P cleavage, and BiP induction. We created a stable cell line expressing ATF6α as well as overexpressing ERp18 (Fig. 6*C*). When a UPR was induced in these cells, the formation of hyperglycosylated ATF6α 467D was dramatically reduced (Fig. 6*D*, compare lanes 2 and 4). In addition, the appearance of ATF6-N was attenuated in this cell line when compared to wild-type (Fig. 6 *E* and *F*, compare 60-min time points). Finally, fold-induction of BiP following activation was reduced upon overexpression of ERp18 (Fig. 6*G*). Taken together, these results indicate a reduction of ATF6α trafficking to the Golgi and processing to generate an active transcription factor following overexpression of ERp18.

## Discussion

The process by which ATF6α becomes activated allowing trafficking to the Golgi is known to involve the dissociation from BiP along with recruitment to transport vesicles (15). Our data presented here demonstrate that this process also involves a change to the redox status of this UPR sensor from a monomer to a specific dimer that is then preferentially trafficked to the Golgi for cleavage by S1/S2 proteases. This redox transition occurs under both chemotoxic and proteotoxic stress, as well as for endogenous and exogenously expressed ATF6α, illustrating independence from the nature of the stressing agent and overexpression. Such a change in redox status potentially distinguishes forms of ATF6α, providing a switch between trafficking or retention. The selective hyperglycosylation of the 467D, combined with exclusively dimeric cleaved ATF6α luminal domain, would attest to the selectivity of this redox switch.

As ATF6α resides in the ER in predominantly an intrachain disulfide-bonded monomer (21), the redox change must involve reduction of the intrachain disulfide as well as formation of a specific interchain disulfide mediated through cysteine 467. Here we propose that the dissociation of BiP from ATF6α leads to the exposure and reduction of the intrachain disulfide by a member of the PDI family. Indeed, previous studies have suggested the exposure of so-called Golgi localization regions following BiP dissociation (34). Formation of the interchain disulfide could then occur by subsequent oxidation. In this scenario, ERp18 could act as a reductase or isomerase functionally interacting with the disulfide-bonded monomer and 467D. Hence, the consequence of ERp18 overexpression would be to restrict dimer formation and lead to a lack of trafficking. In ERp18 KO cells, the 467D would form more rapidly, explaining its faster kinetics to the Golgi apparatus following ER stress. The role of the various PDI family members in facilitating changes to the redox status of individual proteins is likely to be complex due to inherent redundancy (33), but it is clear that only ERp18 associates with ATF6α in the presence of ER stress (16). Hence, while several PDIs may reduce the 467-618 intramolecular disulfide, only ERp18 catalyses the reduction of the 467-467 interchain disulfide. As we have shown that the 467D is preferentially trafficked to the Golgi, the consequence of ERp18 activity will be to regulate the redox transition to this interchain disulfide bonded form and, therefore, Golgi trafficking and ATF6α activation.

The specific trafficking of the 467D to the Golgi raised the question of S1P specificity, which we tested by relocating the protease to the ER. Our results indicate that S1P cleaves both the 467D as well as reduced monomer but cannot cleave the intrachain disulfide bonded monomer or the 618D. Previous studies have shown that S1P can cleave monomeric ATF6α that traffics to the Golgi following treatment of cells with high concentrations of DTT (21). Indeed, we also found that under certain circumstances, such as the C467A mutant, monomeric ATF6α can traffic to the Golgi and be cleaved by S1P. In these instances, the 467-618 intrachain disulfide cannot form. We cannot rule out the possibility that under certain conditions, ATF6α forms noncovalently associated dimers that cannot form the 467 interchain disulfide due, for example, to high concentrations of DTT or the lack of the 467 cysteine. Hence, it may well be the case that dimerization per se is required for trafficking and that this dimer is usually stabilized by the formation of the 467-467 interchain disulfide.

The regulation of Ire1 and PERK by reversible thiol oxidation and the redox-dependent dimerization and trafficking of ATF6α would suggest that redox switches provide a level of regulation for the UPR. Indeed, a change to the ER redox conditions during ER stress has been noted using a redox-sensitive GFP reporter (35) and correct disulfide formation requires an optimal redox balance in the ER (36). While the general mechanism of activation of each pathway remains the dissociation of BiP, it is tempting to speculate that a global change in ER redox could coordinate the response across the three pathways. Hence, the focus of our future work will be to determine how redox changes affect the activation and attenuation of the UPR and whether there are additional factors that are regulated by such redox switches.

## Materials and Methods

### Cell Lines.

All HEK293T, HT1080, and the Flp-In T-Rex doxycycline-inducible HeLa cells were maintained in DMEM supplemented with 10% FBS and 100 units/mL penicillin and 100 µg/mL streptomycin at 37 °C in a 5% CO_2_ incubator.

### DNA Constructs.

The pcDNA 3.1-ATF6α plasmid containing an N-terminal HA-tag and a C-terminal V5-tag followed by a KDEL sequence and stop codon, and the single cysteine to alanine mutations in the luminal domain of ATF6α (ATF6 C467A and ATF6 C618A) have been described previously (16). The pcDNA 3.1/HA-LDLR C646Y has been described previously (27). The human PDIR CXXA substrate-trapping mutant, ERp18 wild-type and ERp18 CXXA substrate-trapping mutant have been described previously (27, 33).

### Cell Culture and Transfections.

HEK293T and HT1080 were either singly or cotransfected with plasmid DNA at 80 to 90% confluence using MegaTran (Origene) transfection reagent. To generate stable overexpression cells, the transfected cells were placed on antibiotic selection for ∼3 to 4 wk until colonies appeared. Screening for positive clones was carried out by Western blotting.

### Cell Lysis, Immunoisolation, and Western Blots.

Cells were harvested by treatment with trypsin-EDTA (Gibco – 25300-054) or by scrapping using a rubber policeman (Greiner bio-one, Cat. No. 541 080). They were collected by centrifugation at 250 × *g* for 5 min and then washed twice with ice-cold PBS (Gibco). The cells were then resuspended in cell lysis buffer (CLB) [1% (vol/vol) Triton X-100, 50 mM Tris⋅HCl, pH 7.4, 150 mM NaCl, 2 mM ethylenediamine tetra-acetic acid (EDTA) and 0.5 mM phenylmethylsulphonyl fluoride (PMSF)], incubated on ice for 10 min, followed by centrifugation at 16,100 × *g* to obtain the postnuclear supernatant. Prior to immunoisolation the postnuclear supernatant was precleared by incubating with protein A Sepharose beads (Generon) for 30 min at 4 °C. The mixture was then cleared by centrifugation at 16,100 × *g* for 1 min and the supernatant incubated with protein A Sepharose beads and the appropriate antibody for 16 h at 4 °C. Immunoisolates were washed three times in CLB. Samples were then boiled at 95 °C for 5 min in 2× Laemmli sample buffer (200 mM Tris-Cl, pH 6.8, 3% SDS, 10% glycerol, 1 mM EDTA, and 0.004% Bromophenol blue) prior to SDS/PAGE either under reducing (treated with 50 mM DTT) or nonreducing conditions.

For Western blotting, proteins were transferred to nitrocellulose membrane (Li-Cor Biosciences), which were blocked in 5% (wt/vol) nonfat dried skimmed milk in TBST [Tris-buffered saline with Tween 20: 10 mM Tris, 150 mM NaCl, pH 7.5, and 0.1% (vol/vol) Tween 20] for at least 60 min. Primary antibodies were diluted in TBST and incubations were carried out for ∼16 h at either 4 °C or at room temperature. Li-Cor IRDYE fluorescent secondary antibodies were used for detection, typically at 1:5,000 dilutions. Blots were scanned using an Odyssey SA imaging system (Li-Cor Biosciences).

### Determination of ATF6α Redox Status and Trafficking during ER Stress.

Confluent HEK/ATF6α cells were pretreated with 30 µM PF429242 (S1Pi) for 60 min before treatment with various ER stressors including DTT (at either 1 mM or 10 mM), 5 µM TG, or 5 µg/mL tunicamycin. Whole-cell lysates were subjected to nonreducing SDS/PAGE, followed by Western blotting. Quantification of band intensities were carried out with the LiCor Image Studio v4 software and histograms prepared using Origin 6.0 professional software.

### Generation of Flp-In T-Rex Doxycycline-Inducible HeLa Cell Line.

The Flp-In T-Rex doxycycline-inducible HeLa/HA-LDLR C646Y cell line was generated by Invitrogen Flip-in T-REx kit according to the manufacturer’s protocol. Briefly, pOG44 and pcDNA3.1/HA-LDLR vector were cotransfected into the cells and selected for by hygromycin B (200 μg/mL) and blasticidin (4 μg/mL). The successfully transfected cells were induced by 2 μg/mL doxycyclin.

### Analyses of ER to Golgi Trafficking of ATF6–O-glycosylation Status.

Cells were treated with 30 µM S1Pi, and either left untreated or treated with 2 mM BADAP for 60 min. The cells were then treated with 10 mM DTT for 30 min. Whole-cell lysates were prepared and incubated either without or with endoH and Peptide: *N*-glycosidase F (PNGase). Samples were separated under reducing SDS/PAGE conditions and ATF6α detected using rabbit anti-HA.

### Sucrose Gradient Fractionation of ATF6α.

HEK/ATF6 cells in 15-cm dishes were either untreated or treated with the S1Pi and ER stressors (5 µM TG for 90 min, 5 µg/mL TM for 2 h). Cells were harvested and washed twice in 1 mL ice-cold PBS/NEM (20 mM NEM, buffer supplemented with EDTA-free protease inhibitor tablet), followed by centrifugation at 1,400 rpm for 5 min. PBS was completely removed, and cell pellet was resuspended in 400 µL of ice-cold buffer A (10 mM Hepes pH 7.4, 250 mM Sucrose, 10 mM KCl, 1.5 mM MgCl_2_, 1 mM EDTA, and 1 mM EGTA) supplemented with protease inhibitor and left on ice for 10 min. Cells were lysed by passing through a 23-gauge needle (Fine-Ject 23G, Henke Sass Wolf) attached to a 1-mL syringe, 20 times, followed by centrifugation at 1,000 × *g*, for 7 min at 4 °C. Next, 200 µL of the resulting supernatant were laid on top a discontinuous sucrose gradient consisting of eight layers, each containing 200 µL of distinct sucrose densities of 20%, 25%, 30%, 35%, 40%, 45%, 50%, and 60%, from top to bottom, in a polycarbonate tubes (11 × 34 mm; Beckman Coulter). The samples were centrifuged at 214,000 × *g* for 60 min at 4 °C, in a TLS 55 rotor using a Beckman Coulter Optima MAX-XP ultracentifuge. Nine fractions of 200 µL each were carefully taken from the top using a pipette. Samples were analyzed on either reducing or nonreducing SDS/PAGE, followed by Western blotting.

### Analyses of ATF6α Cleavage Products.

Cells in 6-cm dishes were either left untreated (−) or treated (+) with either 30 µM S1Pi (60 min), 20 mM ammonium chloride (45 min), 100 nM bafilomycin A1 (4 h), or 5 µg/mL brefeldin A (60 min) treatment, before addition of 5 µM TG to induce ER stress. After treatment, whole-cell lysates were prepared and separated under reducing or nonreducing SDS/PAGE, followed by Western blotting.

### ER Stress-Induced Cleavage of ATF6α.

Wild-type or ERp18 KO HEK293T/ATF6α overexpression cells in 6-cm dishes were either left untreated (−) or treated (+) for at least 1 h with 30 µM S1Pi, before addition of 10 mM DTT or 5 µM TG to induce ER stress. For the brefeldin A (5 µg/mL) treatment, cells were incubated with the drug for at least 1 h prior to harvest.

Isolation and analyses of nuclear ATF6α, ATF6-N, has been described previously (16, 37). Briefly, cells in 6-cm dishes were washed once with 3 mL PBS supplemented with protease inhibitor tablet. Cells were then scraped off the dish in 2.9 mL ice-cold PBS, using a rubber policeman (cell scraper) and transferred into a 15-mL tube. Samples were centrifuged at 1,619 × *g* for 5 min, and supernatant completely removed. The cell pellets were resuspended in 1 mL ice-cold buffer A (10 mM Hepes pH 7.4, 250 mM sucrose, 10 mM KCl, 1.5 mM MgCl_2_, 1 mM EDTA, and 1 mM EGTA) supplemented with protease inhibitor, transferred to 1.5-mL tube, and left on ice for 10 min. Cells were lysed by passing through a 23-gauge needle attached to a 1-mL syringe, 20 times, followed by centrifugation at 1,000 × *g*, for 7 min at 4 °C to obtain the nuclear pellet. The pellet was then resuspended in 100 μL buffer B (10 mM Hepes pH 7.6, 2.5% Glycerol, 420 mM NaCl, 1.5 mM MgCl_2_, 1 mM EDTA, and 1 mM EGTA) supplemented with protease inhibitor, followed by incubation for 60 min on a 4 °C rocker. The samples were centrifuged at 100,000 × *g* for 30 min, 4 °C, to obtain a supernatant containing the nuclear extract. The samples were separated on reducing SDS/PAGE gels, followed by Western blotting.

### Analyses of BiP Induction during ER Stress.

Confluent cells in 6-cm dishes were either left untreated or treated with 1 µM TG for 16 h. Whole-cell lysates were subjected to reducing SDS/PAGE, followed by Western blotting. Quantification of band intensities were carried out with the LiCor Image Studio v4 software and histograms prepared using Origin 6.0 professional software.

### Deglycosylation Assays.

HEK/ATF6 wild-type cells were harvested and washed in PBS then lysed in CLB. Endo H and PNGase reactions were set up according to the manufacturer’s protocol (New England Biolabs). The digests were carried out overnight at 37 °C and samples were resolved on either reducing or nonreducing SDS/PAGE.

### XBP-I Splicing Assay.

Total RNA from TG-treated cells was extracted using TRIzol Reagent (Ambion), according to the manufacturer’s protocol. RT-PCR was carried out using the SuperScript II Reverse Transcriptase (Invitrogen) with oligo dTs (Invitrogen), according to the manufacturer’s protocol. cDNA for endogenous XBP1 was amplified using the following primers pairs: 5′- GAATGAAGTGAGGCCAGTGG -3′, 5′- GGGGCTTCCTATATATGTGG -3′. Actin cDNA was amplified using the following primer pairs: 5′-CCACACCTTCTACAATGAGC-3′, 5′-ACTCCTGCTTGCTGATCCAC-3′. PCR parameters include an initial denaturation step at 95 °C for 5 min; then 35 cycles of 95 °C for 45 s, an annealing step of 60 °C for 45 s, and 72 °C for 45 s; followed by a final elongation step of 72 °C for 10 min.

### Alt-R CRISPR-Cas 9 KO of ATF6.

Using the Integrated DNA Technologies (IDT) Alt-R CRISPR-Cas 9 System, the predesigned guide RNA (gRNA) Hs.Cas9.ATF6.1.AB was selected. We used a single gRNA (cRNA) duplexed with a tracrRNA (IDT, 1072532) and Alt-R S.p. HiFi Cas9 Nuclease V3 (IDT, 1081060) for genome editing. To generate an ATF6 KO, Cas9 nuclease was added to a duplex of 1AB crRNA:tracrRNA in Optimem to form the RNP complex (Fisher, 31985062), which was then transfected into HT1080 cells using Lipofectamine CRISPRMAX transfection reagent (Thermo Fisher, CMAX00001). After 48 h, cells were harvested for genomic DNA using QuickExtract DNA Extraction Solution (Lucigen, QE0905T). Genomic PCR using primers designed for Exon 1 of ATF6α was performed to create template DNA for the T7 Endonuclease assay (New England Biolabs, M0302s). Briefly, the wild-type and 1.AB DNA were hybridized slowly and then digested using T7 endonuclease, to detect any cleavage events by mismatching of the DNA via genome editing events. Samples were analyzed on an agarose gel and by DNA sequencing. After cleavage was confirmed, cells were transferred to 15-cm dishes until colonies appeared (about 10 to 12 d later). Positive ATF6α KO cells were identified by Western blotting using the mouse anti-ATF6 antibody.

## Supplementary Material

Supplementary File

## Data Availability

All study data are included in the main text and *SI Appendix*. All requests for reagents should be directed to the corresponding author.
