# Supplementary material for: Activation of the UPR sensor ATF6α is regulated by its redox-dependent dimerization and ER retention by ERp18
Source: Proc Natl Acad Sci U S A. 2022 Mar 14;119(12):e2122657119. doi: 10.1073/pnas.2122657119 (PMC8944254; doi:10.1073/pnas.2122657119)
Supplement: Supplementary File [file pnas.2122657119.sapp01.pdf]

Supplementary figures

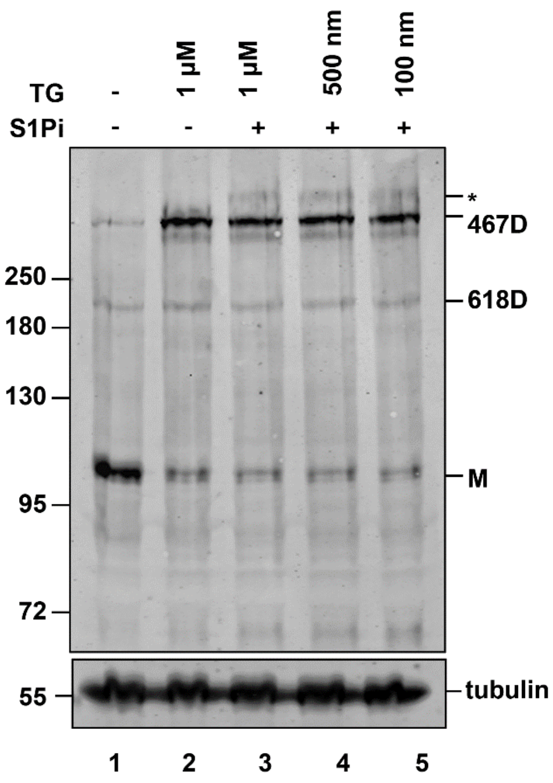

Figure S1: ER stress induction of ATF6α - TG titration

HT1080 cells were either untreated or pre-treated with 30 µM S1Pi for 60 min, and then with various concentrations of thapsigargin as indicated. Samples were separated under non-reducing SDS PAGE conditions and ATF6α detected by western blot. Hyperglycosylated ATF6α is indicated with an asterisk. Western blot of tubulin was used as a loading control.

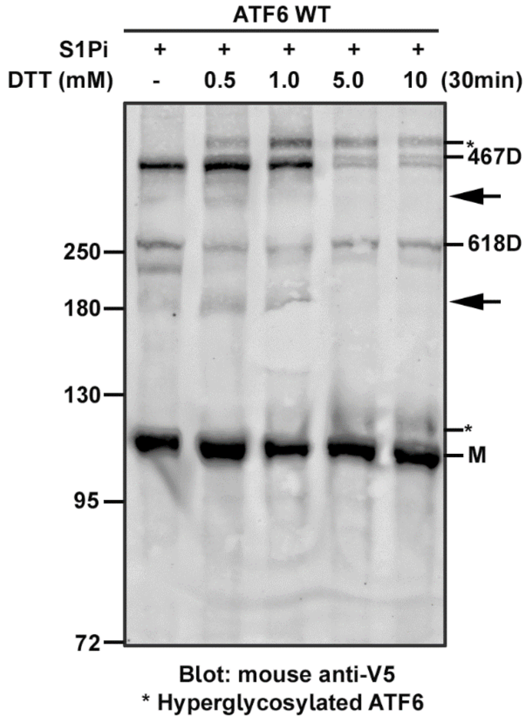

Figure S2: ER stress induction of ATF6α - DTT titration

HEK/ATF6 cells were pre-treated with 30 µM S1Pi for 60 min, and then with various concentrations of DTT as indicated. Samples were separated under non-reducing SDS PAGE conditions and ATF6α detected by mouse anti-V5 western blot. Hyperglycosylated ATF6α is indicated with an asterisk. Arrows indicate uncharacterised V5-reactive bands.

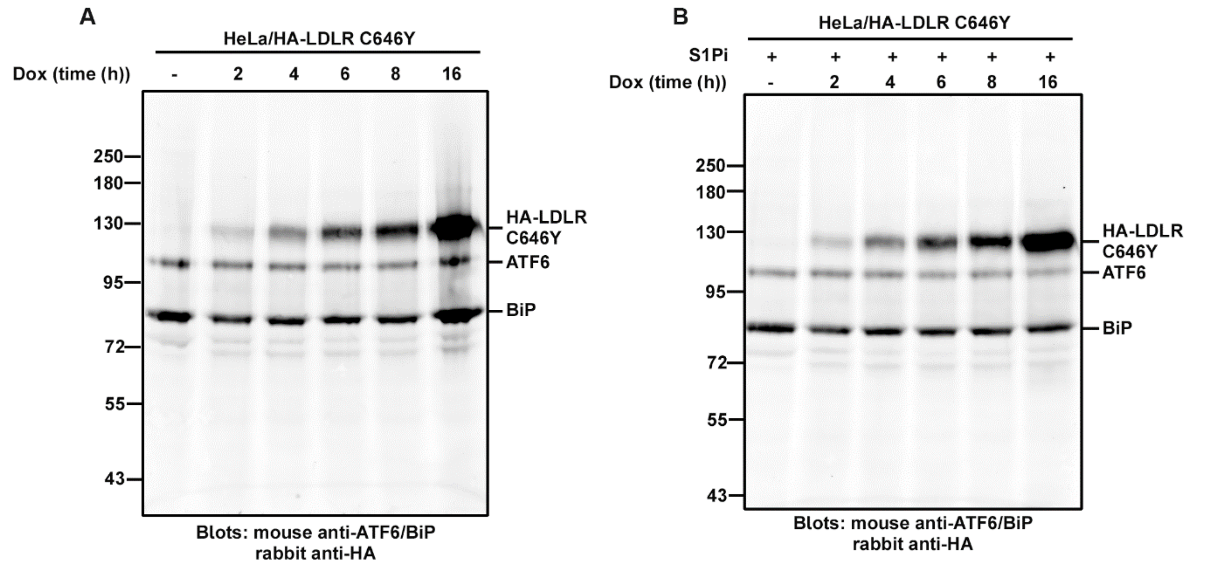

Figure S3: Doxycyclin-induction of HA-LDLR C646Y

Flp-In T-Rex doxycycline-inducible HeLa cells expressing HA-tagged mutant lipoprotein receptor C646Y (HA-LDLR C646Y) were untreated (A) or treated (B) with 30 µM S1Pi for 60 min before treatment with 2 ug/ml doxycycline for the indicated times. Whole cell lysates were separated under reducing SDS-PAGE conditions and immunoblotted with rabbit anti-HA, mouse anti-ATF6α and mouse anti-BiP.

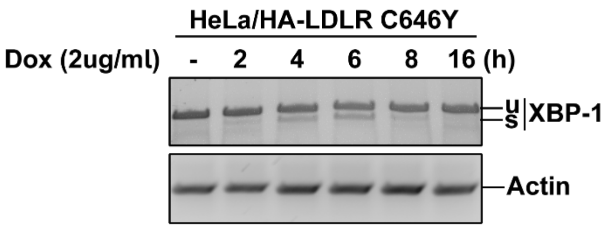

Figure S4: XBP-1 splicing assay

Flp-In T-Rex doxycycline-inducible HeLa cells expressing HA-tagged mutant lipoprotein receptor C646Y (HA-LDLR C646Y) were treated with 2 ug/ml doxycycline for the indicated times. RT-PCR analysis was carried out with oligos specific for XBP-1. PCR products were resolved by gel analysis to determine the spliced (S) and unspliced (U) forms of XBP-1. Actin was used as a control.

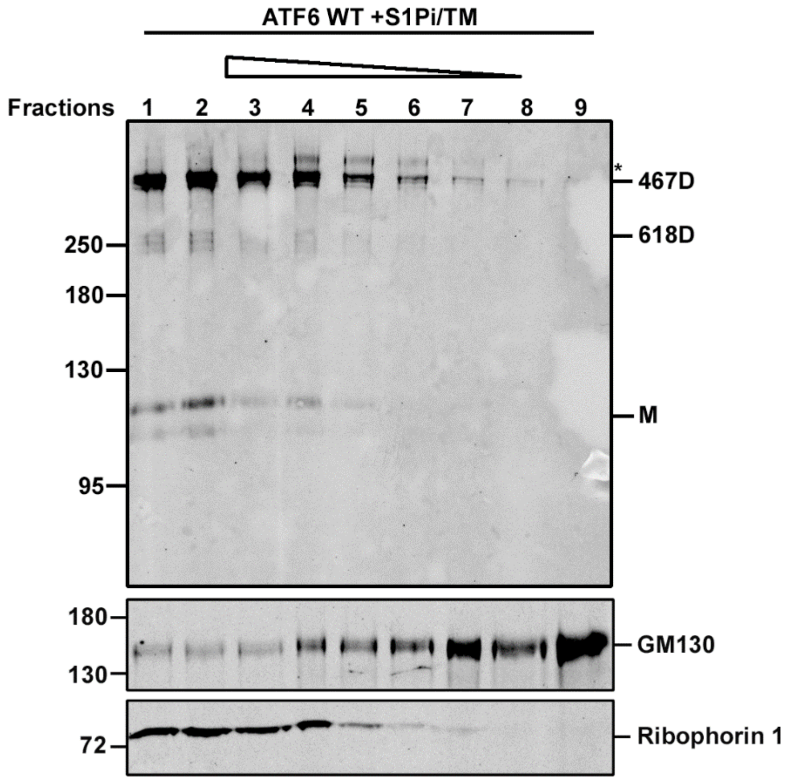

Figure S5: Sucrose gradient fractionation of tunicamycin-treated HEK/ATF6 cells

HEK/ATF6 cells were pre-treated with 30 µM S1Pi for 60 min, and then with 5 µg tunicamycin (TM) for 90 min before sucrose gradient fractionation. Fractions were separated under non-reducing SDS PAGE conditions and ATF6α detected by rabbit anti-HA western blot. Ribophorin I and GM130 were detected with rabbit anti-ribophorin I and rabbit anti-GM130 respectively. Hyperglycosylated ATF6α 467D is indicated with an asterisk.

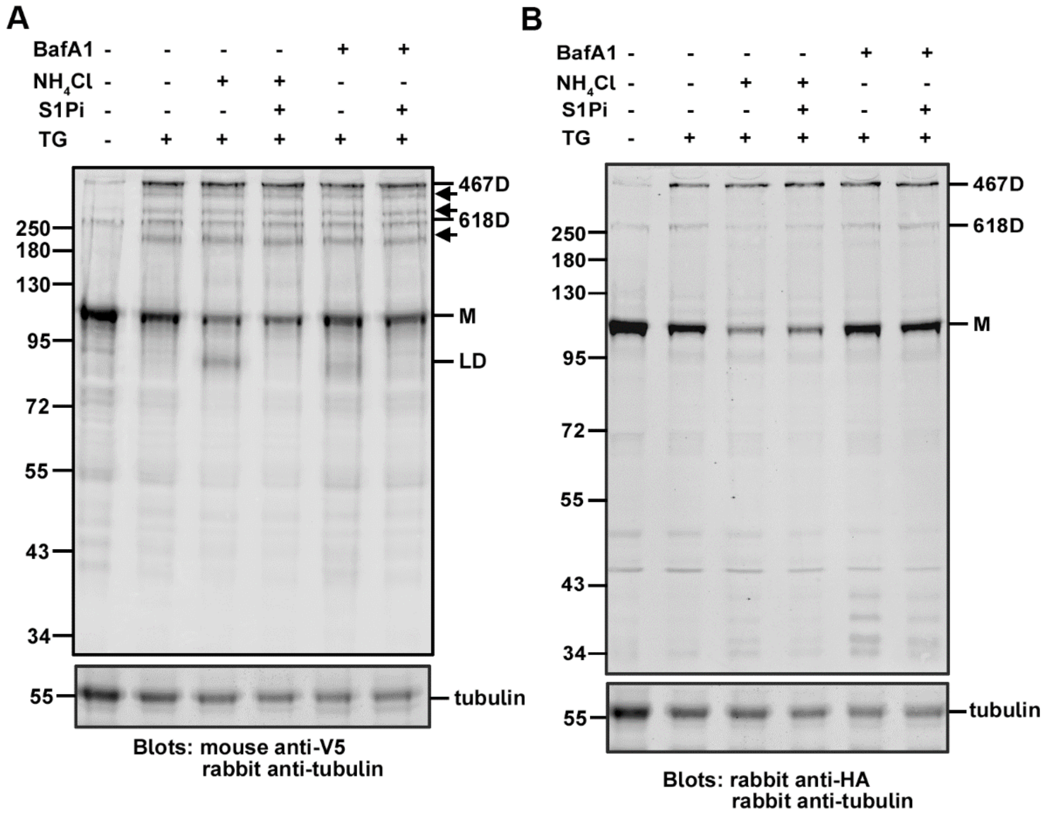

Figure S6: ER stress induced cleavage of ATF6α

(A-B) HEK/ATF6 cells, were either untreated (A) or treated (B) with 500 nM bafilomycin A1 (BafA1) for 8 h, 20 mM ammonium chloride (NH_4Cl) for 45 min, and then with or without 5 µM TG, for the 60 min. Cell lysates were separated under non-reducing SDS-PAGE conditions and ATF6α detected with mouse anti-V5 (A) or rabbit anti-HA (B). Tubulin was used as a loading control.

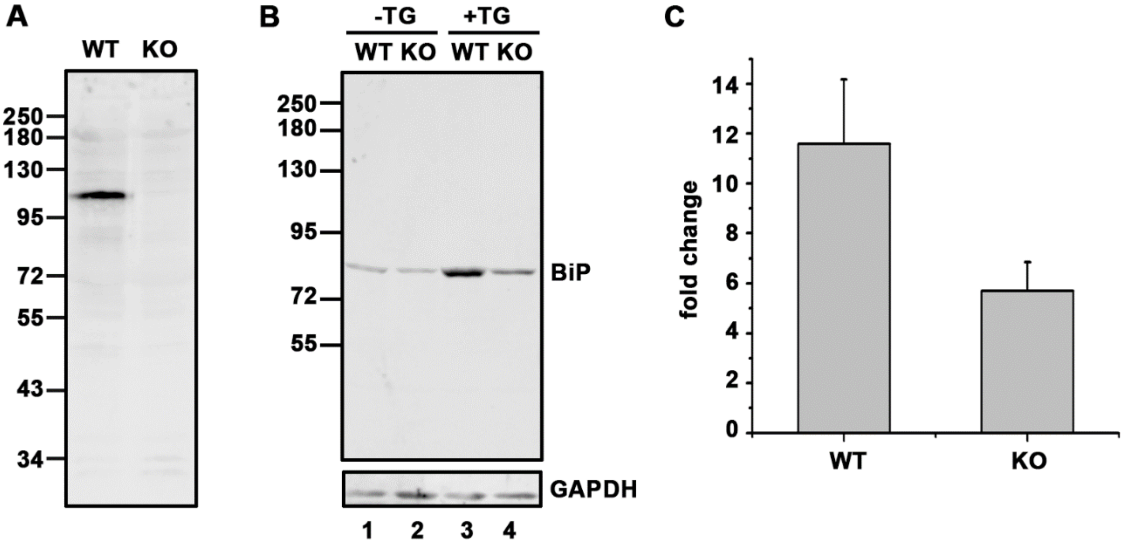

Figure S7: Deletion of ATF6 in HT10180 mitigates BiP induction during ER stress

(A) Western blot of cell lysates from HT1080 wild type (WT) and ATF6α KO cells with anti-ATF6 verifying deletion of ATF6α.

(B) Wild type (WT) or ATF6 KO cells (KO) were left untreated (-TG) or treated with 1 µM TG (+TG) for 16 h. Lysates were separated under reducing SDS-PAGE and levels of BiP and GAPDH detected by immunoblot analysis.

(C) Quantification of BiP levels from three experiments, error bars represent ± SD.
